# Consistent nonlinear optical refractive index $n_2$ measurement of porcine crystalline lens and its surrounding in the 650–900 nm range

**DOI:** 10.1038/s41598-026-48802-x

**Published:** 2026-04-24

**Authors:** Vincent Comte, Julien Bilal Zinoune, Tom Meunier, Philippe Gain, Gilles Thuret, Florent Bessin, Christophe Cassagne, Georges Boudebs, Cyril Mauclair

**Affiliations:** 1 BiiO, Biology, Engineering and Imaging for Ophthalmology Laboratory EA2521, Faculty of Medicine, Saint-Etienne, France; 2 KERANOVA SA, Saint-Etienne, France; 3 LPHIA, SFR MATRIX EA4464, University of Angers, Angers, France

**Keywords:** Nonlinear refraction, Aqueous humor, Eye lens, Vitreous humor, Phase object, Medical research, Optics and photonics, Physics

## Abstract

The nonlinear optical properties of transparent ocular media have been shown to alter the focused intensity distribution of femtosecond laser pulses, potentially affecting the precision of laser ophthalmic surgery. In this study, the nonlinear refractive index $n_2$ of porcine aqueous humor, crystalline lens and vitreous humor was measured using two complementary techniques: the standard Z-scan-derived D4$\sigma$ method and the phase-object imaging method. Both were performed with a tunable femtosecond laser source across the visible to near-infrared range (650–900 nm). All measured $n_2$ values were found to be close to $2 \times 10^{-20}\,m^2/W$ within experimental uncertainties, with no measurable nonlinear absorption detected. Notably, the phase-object technique proved particularly well-suited for slightly scattering or heterogeneous media, such as the freshly extracted crystalline lens, when calibrated against a reference medium (here, water). These experimental results allow for a quantitative numerical assessment of pulse and beam degradation due to nonlinear refraction during procedures like cataract surgery, as well as an evaluation of its potential impact on photodisruption geometry.

## Introduction

Thanks to their high structuring precision inside transparent media, femtosecond laser pulses have revolutionized several ophthalmic surgery procedures^1,2^. Relying on highly nonlinear (NL) energy coupling phenomena such as the multiphotonic ionization, the ultrafast laser energy can be deposited within a micrometric focal volume. This allows for a well-controlled photodisrupted volume upon beam displacement inside the transparent tissue such as the cornea or the crystalline lens with a reduced amount of thermal effects^3^. In that frame, a precise knowledge and control of the laser intensity distribution within the focal region is of upmost importance as the geometry and regularity of the excitation volume heavily depends on the laser beam shape, focusing and aberrations^4^. Moreover, ultrafast laser pulses propagating in transparent media are prone to undergo NL effects that can lead to beam profile distortion, uncontrolled displacement of the focal region and spatio-temporal defacement of the pulse^5^. Even slight beam or pulse irregularities can lead to strong alterations of the laser-matter interaction in the presence of nonlinearities. This could render the use of ultrafast laser pulses unadapted to precise eye surgery. Well-known examples of such phenomena include self-focusing^6^ and self-phase modulation that can alter the laser beam concentration as well as its spectral and temporal characteristics. Prior to photodisruption, other highly unstable effects can occur. They are related to the onset of excited carriers and their optical properties that also have an effect on the beam convergence. This can lead to the so-called filamentary propagation that relies on an interplay between self-focusing and plasma defocusing^7,8^.

These unsteady effects are to be carefully handled for eye surgery. The tight focusing and relatively long pulse duration - $\approx$ 330 fs at Full Width Half Maximum (FWHM) used in that case - strongly limits this focusing-defocusing competition, allowing for an efficient light concentration along the optical axis^9^. However, when aiming at deep ophthalmic tissues such as the bottom of the crystalline lens or even the retina, the geometrical focusing power is limited by the maximum eye conical angle. Thus, filamentary-like behavior should not be ignored even in relatively tight focusing conditions, as observed by Sudrie et al. in fused silica^10^. This resonates particularly with the case of laser cutting in ophthalmic surgery using ultrafast pulses^1^, especially now that spatial beam techniques are involved^11^. Indeed, studying the behavior of advanced beam shapes undergoing NL propagation effects is a complex task that requires a fine knowledge of the propagation medium NL properties^12^.

Consequently, there is strong interest in measuring the NL properties of the main eye constituents with the highest degree of precision using state-of-the-art techniques. The Z-scan is a well-known method that allows for an accurate measurement of NL absorption $\beta$ and NL refractive index $n_2$ of transparent media^13,14^. While several improvements have been proposed^15^, two techniques that derive from the pioneering work of Sheik-Bahae et al.^13^ are of particular interest, namely the D4$\sigma$^16^ and the phase object^17^ imaging method. It has recently been shown to be a cost-effective method for pulse duration determination, thanks to its good precision^18^. The D4$\sigma$ technique, also referred to as Beam Size Relative Variation (BSRV), is characterized by its high precision in determining the NL refractive index $n_2$, with minimal sensitivity to the laser beam profile. Notably, it enables a direct measurement of the absolute value of $n_2$, making it suitable for quantitative NL optical analysis. Concerning the phase object imaging method, it has the advantage of not requiring any displacement of the sample. As a single-shot laser technique, it simplifies the experimental setup while ensuring a reasonable level of measurement reliability^19^. It is therefore better suited for slightly diffusive media, particularly those lacking parallel surfaces and exhibiting internal refractive index inhomogeneities, as further discussed below. Thus, the need for quantitative characterization of NL properties of the eye constituents for precise control of the interaction zone^6,20^ can be met by these two $n_2$ characterization techniques.

In this paper, we report on the measurement of the NL refractive index $n_2$ of porcine aqueous humor, crystalline lens, and vitreous humor using the D4$\sigma$ and the phase object imaging techniques with an accordable femtosecond laser source (Optical Parametric Amplifier - OPA) for the 650-900 nm spectral range. We then numerically evaluate the pulse and beam propagation distortion due to the NL self-focusing and self phase modulation effects in the anterior eye segment.

## Results

**Fig. 1:**
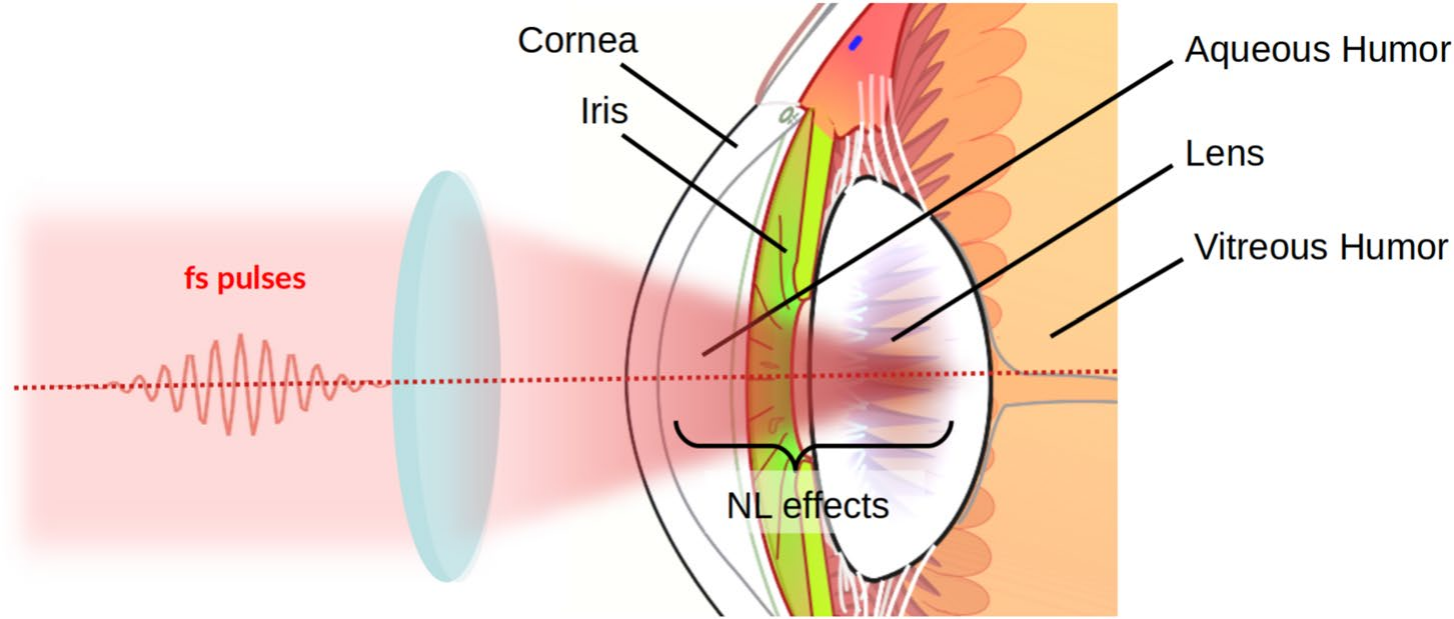
Scheme of the human eye anterior segment under femtosecond laser exposure for crystalline lens surgery. NL effects can occur before the focused laser spot, i.e, in the cornea, the aqueous humor, the lens and even the vitreous humor in the case of retinal surgery. Adapted from^21^ under the Creative Commons Attribution-Share Alike 3.0 Unported license.

A scheme of the anterior segment of the human eye is presented in Fig. 1. During eye surgery, such as the femtosecond assisted cataract surgery (FLACS)^22^ or FemtoMatrix® treatment^11^ as well as in vitro-retinal laser surgery^23^, the laser beam passes through several layers of biological tissues and liquids: successively the cornea, the aqueous humor, the crystalline lens and the vitreous humor. All these media may induce NL beam distortions within the laser interaction zone. Nextly, we experimentally measure the NL refractive index $n_2$ value for the lens, the aqueous and the vitreous humors using two techniques, namely the D4$\sigma$ and the phase object imaging technique (see the Methods section).

The measurements are performed across the 650–900 nm spectral range for two primary reasons. First, early ophthalmic surgery lasers used the available Ti:sapphire laser systems at 800 nm wavelength^1^. Investigations within the near-infrared spectral region even below 1 $\mu$m were followingly conducted^24^ while recent studies still employ the 800 nm wavelength to address glaucoma as proposed by Liu et al.^25^. Thus, there is significant scientific and clinical interest in exploring these spectral regions for both surgical and diagnostic applications, where knowledge of $n_2$ is essential. Second, and more importantly, this range lies within the transparency window of the main ocular media^26^, where no significant linear or nonlinear absorption is expected. Verifying that $n_2$ remains constant and that nonlinear absorption is negligible across this interval not only confirms the absence of resonant enhancement but also strengthens the reliability of the measured $n_2$ value and supports its use for modelling at nearby wavelengths, including the 1 $\mu$m region used in clinical practice. Moreover, ophthalmic tissues ablation threshold are known to vary with respect to the laser wavelength^27^. In this work, we neglect the effect of the lens capsular bag due to its low thickness ($\approx 10\,\mu m$).

### n$_2$ measurements using the D4$\sigma$ technique

**Fig. 2:**
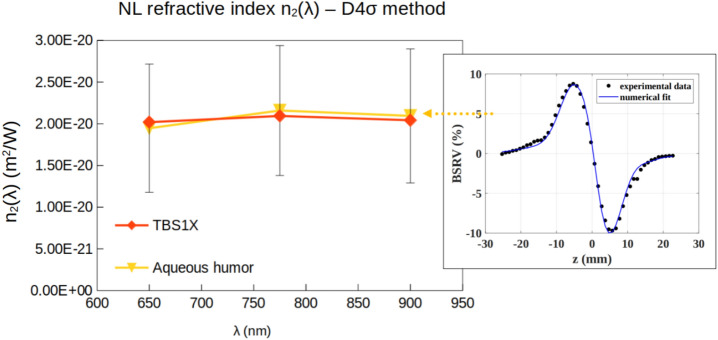
Nonlinear refractive index $n_2$ experimental measurements within the 650–900 nm spectral range using the D4$\sigma$ method for the aqueous humor and the TBS1X solution. A typical Beam Size Relative Variation (BSRV) Z-scan trace is shown on the right with the beam size measurement data points (black) and the fitted curve (blue).

A first series of measurements was conducted using the D4$\sigma$ technique, which is a derivation of the Z-scan technique as explained in the Methods section. The investigated solutions were the TBS1X solution and the aqueous humor. The details concerning the TBS1X solution composition can also be found in the Methods section. The results are shown on Fig. 2 with a typical Z-scan type curve. The variation of the beam size (i.e, the shape and amplitude of the BSRV curve evaluated using a numerical fit) permits to experimentally evaluate the NL refractive index value. We note that this quantity is remarkably constant over the spectrum as depicted on Fig. 2. Indeed, the standard deviation across 6 measured values is only $4 \times 10^{-22}\,m^2/W$. Therefore, the n$_2$ value was found to be $2.1 \pm 0.4 \times 10^{-20}\,m^2/W$ on average on the entire 650-900 nm spectrum for both solutions. The D4$\sigma$ technique requires optically clean, transparent and homogeneous samples with very low diffusivity. Indeed, scattering and inhomogeneities within these tissues directly alter the beam profile during sample translation, resulting in noisy BSRV curves. Under these conditions, the underlying mathematical framework required to deduce $n_2$ is no longer valid. Consequently, the $n_2$ values for the remaining solutions and tissues were characterized using the phase object imaging method, which is an imaging single shot measurement, less sensitive to z-scanning perturbations.

### $n_2$ measurements using the phase object imaging technique

**Fig. 3:**
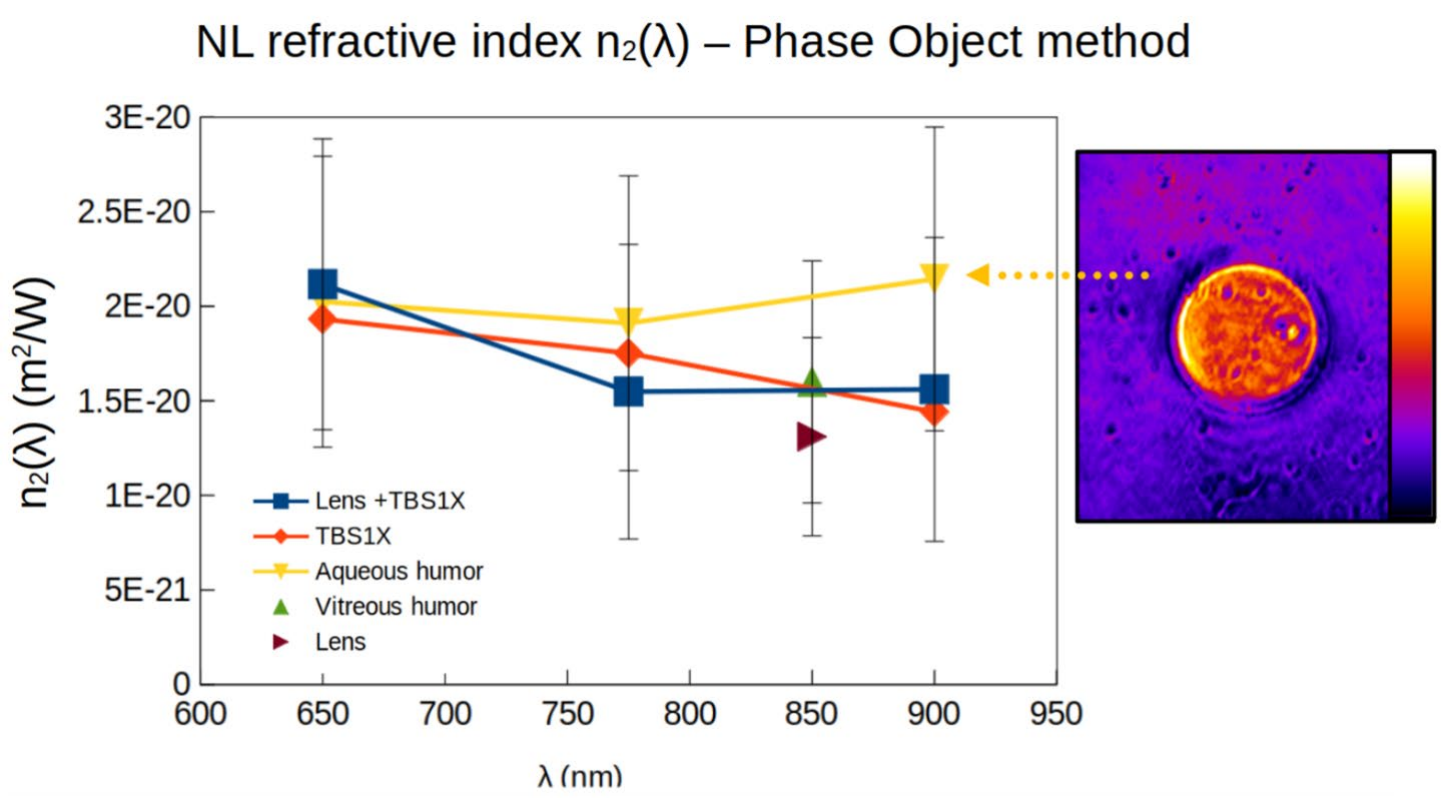
Nonlinear refractive index $n_2$ experimental measurements within the 650–900 nm spectral range using the Phase object imaging method for aqueous humor, vitreous humor, TBS1X, TBS1X with diluted lens and the crystalline lens. An example of the phase object image from which the contrast is measured is shown on the right with the colorbar representing arbitrary intensity units and used for visualization purposes only.

The $n_2$ of aqueous humor, vitreous humor, TBS1X, TBS1X+lens (with diluted lens) and the lens alone were measured using the phase object imaging method^17^. The results are shown on Fig. 3 left, with a typical image of the phase object (right). As explained more in detail in Methods section, the n$_2$ value can be calculated from the contrast between the background and the circular phase object region. Concerning the aqueous humor, an averaged value of $2.0 \pm 0.8 \times 10^{-20}\,m^2/W$ with a standard deviation of $1.2 \times 10^{-21}\,m^2/W$ is found across the investigated spectrum, which is consistent with the previous result. A similar observation applies to the TBS1X solution, which yielded an average $n_2$ value of $1.7 \pm 0.8 \times 10^{-20}\,m^2/W$ (standard deviation: $2.5 \times 10^{-21}\,m^2/W$). The TBS1X solution with lens exhibited an identical average value and uncertainty. We observe a slight decrease in $n_2$ with increasing wavelengths (see Fig. 3), a trend that follows the dispersion of the linear refractive index. However, the magnitude of the experimental uncertainty precludes a definitive conclusion on this supposed $n_2$ wavelength dependence. For the vitreous humor, $n_2$ was determined at 850 nm to be $1.6\pm 0.8 \times 10^{-20}\,m^2/W$, a value comparable to that of the crystalline lens, measured as $1.3\pm 0.8 \times 10^{-20}\,m^2/W$. Due to the increased complexity of handling these ex-vivo tissues and their susceptibility to optical degradation over time, comprehensive wavelength-dependent measurements for the crystalline lens and vitreous humor were not feasible. The measurement at 850 nm was therefore chosen as a representative point within our spectral window. Given the weak wavelength dependence observed for the other, more stable media (aqueous humor and TBS1X solutions) across the entire 650-900 nm range, and the consistent $n_2$ values found for all media within the experimental uncertainties, it is reasonable to consider that the $n_2$ of the lens and vitreous humor is also on the order of $2 \times 10^{-20}\,m^2/W$ across the whole spectrum.

Overall, the $n_2$ values measured across the 650–900 nm spectral range are consistently on the order of $2 \times 10^{-20}\,m^2/W$. Given these quantitative results, we now evaluate the resulting beam and pulse deterioration in the context of femtosecond laser-assisted surgery within the crystalline lens.

### Beam and pulse deterioration when focused under nonlinear conditions

#### Spectral and temporal effects

In the following, we briefly evaluate NL propagation effects related to the measured $n_2$ value for typical cataract surgery laser parameters. We thus consider a Gaussian pulse of energy $E = 1~\mu \text {J}$, FWHM duration $\tau = 330~\text {fs}$, and spectral width $\Delta \lambda _0 = 4.7~\text {nm}$, centered at $\lambda _0 = 1030~\text {nm}$ without any chirp. The beam is focused down to a waist $w_0 = 3\,\mu m$. These parameters fall entirely within the range of typical nowadays femtosecond laser assisted cataract surgery machines^22^. With these values, and neglecting any NL beam or pulse distortions, it is possible to evaluate the laser spatial and temporal peak central intensity $I_0$ at focus using:

$$I_0 \approx 2 \cdot \frac{E}{\tau \cdot \pi \cdot w_0^2} \approx 2.14 \times 10^{13}\,W \cdot cm^{-2}$$

Note that we use a value factor of 2 by combining factors of Fundamental Gaussian spatial profile (equal to 2), and temporal gaussian profile (equal to 1.06). Let us consider the NL phase shift $\Delta \phi$ occurring within the focal region, where the beam intensity is highest. It can be expressed as $\Delta \phi = n_2\cdot I_0\cdot d \cdot {2\cdot \pi } /{\lambda _0}$ where *d* is the effective propagation length over which the NL retardance accumulates. An upper bound for the NL phase shift $\Delta \phi _{max}$ can be estimated by assuming that the beam intensity remains constant over the propagation distance equal to the Rayleigh length in medium $Z_R = d = {\pi \cdot w_0^2\cdot n} /{\lambda _0}$, where $n \approx 1.4$ is the linear refractive index of the crystalline lens near ${\lambda _0}$^28,29^. Subsequently, $Z_R \approx 38.4 ~\mu m$. Using parameters discussed previously, $\Delta \phi _{max} = 1.00~\text {rad}$. The corresponding worst-case spectral broadening due to self-phase modulation (SPM) can then be estimated using the analytical Agrawal’s formulae^30^:

$$\Delta \lambda _\text {SPM} \approx 0.86 \cdot \Delta \phi _\text {max} \cdot \Delta \lambda _0 \approx 4.04~\text {nm}$$

With this enlarged spectrum, an upper bound of the laser pulse elongation $\Delta \tau _{NL}$ due to SPM over $Z_R$ can be calculated using the experimentally measured group velocity dispersion of the cornea-lens complex $\beta _2 \approx 33\,fs^2/mm$ (at 800 nm)^31^ following:

$$\Delta \tau _{NL} \approx \beta _2 \cdot Z_R \frac{2 \cdot \pi \cdot c}{\lambda _0^2}(\Delta \lambda _0 +\Delta \lambda _{SPM})$$

The calculation reveals $\Delta \tau _{NL}\approx 2 \times 10^{-2}~\text {fs}$ which is clearly negligible when compared to the initial pulse duration. Likewise, the pulse elongation due to linear GVD is also found to be negligible even when considering the total laser path length down to the retina. In that case, the related chromatic dispersion, or group delay dispersion (GDD) was measured by Coello et al.^31^ to be $\phi ''=665\,fs^2$ at 800 nm. Thus, the corresponding elongated pulse duration $\tau '$ due to linear GDD can be calculated following:

$$\tau '= \tau \cdot \sqrt{1+\left( \frac{4\cdot \phi '' \cdot ln(2)}{\tau ^2}\right) ^2}$$

which yields $\tau ' \approx 330.05\,fs$. By considering the main linear and NL pulse elongation phenomena, we can conclude here that typical eye surgery laser parameters to address the crystalline lens do not lead to significant deterioration of the pulse duration, and consequently low threshold variation effects are to be expected in relation to these phenomena. We have further verified this result using a split-step algorithm programmed under Python, readily available on the Github open-source repository PyLUMINA (scenario 16)^32^.

It should be noted that our measurements and calculations pertain to clear optical media–specifically, a non-cataractous crystalline lens. Pathological conditions such as cataract or corneal disease introduce significant optical scattering and absorption^24,33^, which in practice necessitate adjustments to the laser pulse energy to achieve effective photodisruption. However, the present analysis indicates that, for the experimentally measured $n_2$ values, NL spectral and temporal broadening effects should have negligible impact on the peak power intensity attained at the intended photodisruption plane within the lens. We now examine the effects on the spatial beam distribution.

#### Spatial effects

**Fig. 4:**
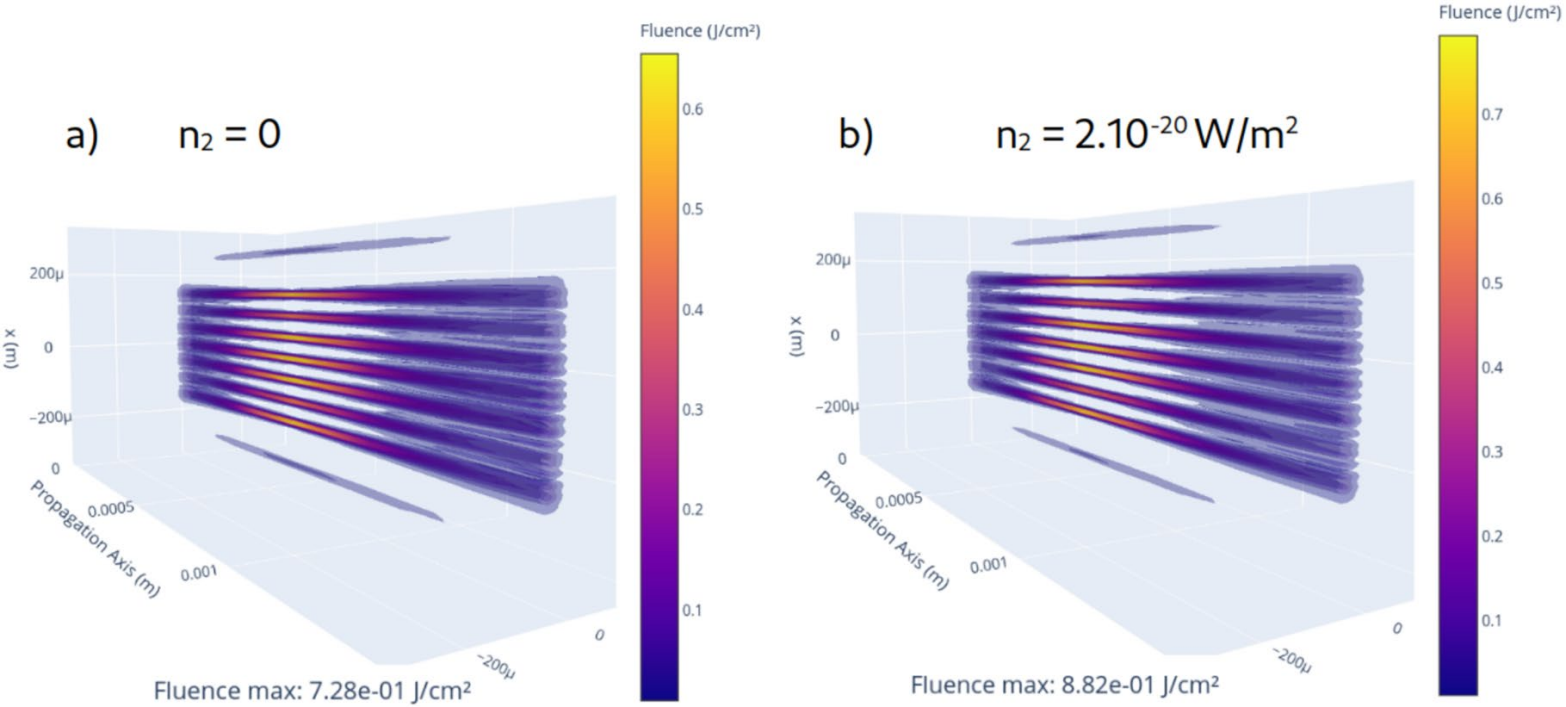
Simulation of multi-spot beam propagation without (**a**) and with (**b**) the nonlinear $n_2$ spatial contribution. The simple split step model permits to anticipate a slight laser fluence increase in the presence of nonlinearity.

The NL refractive index $n_2$ also has well-documented consequences for beam propagation, most notably the self-focusing effect. Here, a NL spatial phase retardance $\phi _{NL}$ modifies the beam profile according to the intensity distribution: $\phi _{NL} \propto n_2 \cdot I(x,y)$. For a Gaussian beam, this phase modulation effectively acts as a converging lens, further reducing the beam waist along the propagation axis and potentially leading to catastrophic self-focusing (beam collapse). This phenomenon is harnessed in Kerr-lens mode-locking of femtosecond oscillators to favor the amplification of intense ultrashort pulses^34^ and also forms the basis of Z-scan techniques for measuring $n_2$. In the context of focusing ultrafast pulses inside transparent materials, however, self-focusing introduces instabilities in the final focal position^10^. The risk of beam collapse can be assessed via the critical power $P_{CR}$, at which diffraction balances self-focusing. For a Gaussian beam, $P_{CR}$ is given as^35^:

$$P_{CR} = 1.9 \cdot \frac{\lambda ^2}{4\cdot \pi \cdot n_0\cdot n_2}$$

With the experimentally measured $n_2$, $P_{CR}$ reaches 6.2 MW which is 3 times the power $P_0$ carried by a $E=1$ $\mu$J laser pulse. The effect should be negligible for a single Gaussian beam. We can further evaluate the associated focal shift $z_f$ following Marburger’s formulae^36^:

$$z_f = \frac{0.367 \cdot \pi \cdot n_0\cdot w_0 ^2/\lambda _0}{\sqrt{\left( \sqrt{P_{CR}/P_0}-0.852\right) ^2}-0.0219}$$

Considering the parameters discussed, $z_f \approx 18\,\mu m$ which constitutes a non-negligible fraction of the Rayleigh length $Z_R$. However, this shift in the effective focal position is negligible compared to the typical focal length of the focusing lens, which is on the order of several millimeters. Furthermore, safety margins exceeding $100\,\mu m$ are routinely employed when positioning the laser focal spot within the cornea or crystalline lens, aided by real-time optical coherence tomography (OCT) monitoring of photodisruption effects^11^.

If the situation seems quite clear for a regular Gaussian beam, let us investigate the case of a spatially-shaped beam distribution focused inside the crystalline lens^11^. To do so, we developed a Fourier-based non paraxial propagation code based on the split step approach in order to include the spatial NL $n_2$ effects under Python readily available on the GitHub open-source repository PyLUMINA (see scenario 15)^32^. As depicted on Fig. 4, a seven spot beam distribution undergoes some modifications when the $n_2$ contribution is included in the laser propagation. The maximum attained fluence appears to be slightly higher in the presence of $n_2$ but the overall focusing geometry remains very similar. Further calculations could be conducted, to better include the electronic response (plasma defocusing) as well as temporal effect by solving the NL Schrödinger Equation, however this is outside the scope of this paper.

## Discussion

A key finding of this study is that the $n_2$ values for all tested ocular media–aqueous humor, vitreous humor, and the crystalline lens–are, within experimental uncertainty, very close to the well-established value for water ( $\approx 2 \times 10^{-20} m^2/W$)^37,38^. The slightly lower average values observed for the TBS1X buffer ($1.7 \times 10^{-20} m^2/W$) and the crystalline lens ($1.6 \times 10^{-20}m^2/W$) fall well within the combined uncertainties of the two measurement techniques and the biological variability of the samples. Therefore, we do not interpret these minor variations as evidence of a significant compositional effect. Instead, the primary conclusion is that water is the primary determinant of the ultrafast nonlinear optical response in these ocular media. This finding is consistent with the high water content of these tissues. Consequently, for the purpose of modeling nonlinear propagation in a clear lens, approximating its $n_2$ with that of water is a valid and practical approach.

The dominant NL effect expected in ocular media under femtosecond irradiation is Kerr self-focusing, while self-phase modulation may also occur and slightly modify the laser spectrum and pulse duration. However, for Yb-based femtosecond sources used in ophthalmic surgery–typically delivering 300–400*fs* pulses with moderate peak powers, the SPM-induced temporal broadening remains negligible. Linear dispersion effects are also insignificant: measurements of the group velocity dispersion (GVD) in the cornea, crystalline lens and vitreous humor show that, although the cornea and lens are more dispersive than water by factors of $\sim 5$ and $\sim 2$ respectively, their limited physical thickness contributes only weakly to the overall chromatic spreading across the axial length of the eye^39^. Consequently, both linear and NL temporal distortions are expected to remain minimal under standard cataract-surgery focusing conditions which is consistent with our calculations.

From a spatial point of view, our experimentally determined NL refractive indices $n_2$ confirms that self-focusing is weak for clinically used pulse energies, and does not significantly distort the focal geometry in homogeneous media. This aligns with earlier studies reporting negligible NL effects in the cornea for comparable conditions^40,41^. Our simulations do not exhibit significant Kerr-induced modification in settings analogous to those of the human eye. Nevertheless, future modeling efforts based on the NL Schrödinger equation may be relevant for advanced beam shapes, including vortex beams recently explored for corneal cutting and showing promising improvements in incision quality^42^.

Beyond ultrafast Kerr effects, thermal lensing constitutes another potential source of beam distortion. Although negligible for low-duty-cycle femtosecond surgery systems, thermal effects are known to dominate in Q-switched Nd:YAG regimes, where each pulse induces a transient defocusing lens in ocular tissues with relaxation times of several seconds^43^. With the rapid increase of repetition rates in modern femtosecond surgical systems (100 kHz and above), heat accumulation becomes a realistic concern, as observed in glass where MHz-rate femtosecond pulses generate significant thermal buildup and permanent refractive modifications^44^. Sensitive single-beam measurement techniques have demonstrated the capacity to resolve very weak thermal and Kerr contributions^45^, highlighting the need for future quantitative studies of thermal lensing in biological ocular media at high repetition rates. Moreover, we have simplified the $n_{2}$ characterization using an homogeneous medium for this study. However, it is known that the crystalline lens is not homogeneous. In particular, it presents a linear refractive index gradient depending on the position in the lens and the patient age^46^. Further investigations should be conducted to take that into account; ideally performing $n_{2}$ measurements on various portions of the crystalline lens and various patient age. We stayed focused on aqueous humour, lens, and vitreous humour because of the existence of prior research for the determination of NL index of corneas using the Z-scan technique, measured to be $2 \times 10^{-19}\,m^2/W$ for porcine cornea^40^ and $1.6\pm 0.2 \times 10^{-19}\,m^2/W$ for human cornea with a water content of 78 %^41^.

## Methods

### Experimental apparatus

For this experiment, we used a Pharos femtosecond laser source and Orpheus collinear optical parametric amplifier (OPA), with an emitting wavelength centered at 650 nm, 775 nm or 900 nm depending on the study case (Pharos with Orpheus OPA, Light Conversion, Lithuania) on a “quasi-4f” coherent imaging system (using one converging lens of f=25 cm). A known phase object is used for the phase object imaging technique. Samples are encapsulated in a 10 mm useful thickness cuvette (12.5 mm total thickness, synthetic quartz, ref CV10Q355, Thorlabs, USA).

A Glan-Taylor calcite polarizer (GT10-Thorlabs) was placed after the OPA to ensure clean linear polarization and precise intensity control. As more precisely described in^18^, the laser pulse duration was precisely measured by a home-made autocorrelator apparatus based on a 2 photon photodiode (model EPD-440-02.5). The laser pulse duration was measured to be 330 fs and the emission of single pulses was synchronized to the camera image acquisition (cooled-CMOS Kiralux DC126MU Thorlabs).

Two NL index measurement techniques are used here, namely the Phase object imaging technique and the D4$\sigma$ technique. Both techniques are based on a similar apparatus involving a pseudo 4f-imaging scheme with a converging lens of $f=25\,cm$ (see details below). The Pharos femtosecond laser system was operated at a repetition rate around 0.5 Hz. This low repetition rate ensures that the time interval between successive pulses (2 seconds) is much longer than the thermal diffusion time constant in the aqueous samples, thereby preventing the accumulation of heat and eliminating any potential contribution from thermal lensing to the measured nonlinear signal.

To ensure that the measured nonlinear response was due to the intrinsic Kerr effect and not to laser-induced damage, we implemented several control measures. For all samples, we verified the absence of nonlinear absorption. Furthermore, during Z-scan measurements, we continuously monitored the beam profile for any irreversible distortion by comparing images taken before and after the sample translation. There was no data showing signs of permanent modification. These precautions, combined with the low repetition rate used, give us confidence that our measurements reflect the true $n_2$ electronic response of the materials.

### Sample preparation

We obtained 6 months old porcine eye globes from a local slaughterhouse, enucleated immediately after sacrifice, and kept at room temperature. The sample preparation was carried out within 24 hours. Aqueous and vitreous humors were extracted directly from porcine eye globes, through the cornea, using sterile needles. Crystalline lenses were extracted from eye globes using surgical tools. Only clear lenses were selected, and processed immediately following their extraction. Two types of sample preparation were conducted for the lens.

The first procedure permitted to obtain a liquid solution easily poured inside the quartz cuvette. As crystalline lenses are not homogeneous, the lens underwent several preparation steps in order to obtain an homogeneous solution from them. We tried four methods close to those used for protein extraction and analysis. The concentration was within the $680-750\,mg/mL$ range for all the solutions:

1. The first method consisted in a simple dilution in pure water followed by a mechanical homogenization.
2. The second one used a Tris-HCl solution with a pH of 8.5 as described by Truscott et al.^47^.
3. The third one was based on an extraction buffered of Tris-HCl and ethylenediaminetetraacetic acid as described by Giurgola et al.^48^.
4. The last one is a custom-made method developed in the BiiO laboratory, which involves the use of a buffer solution adjusted at a pH of 7.4 with 1 % Sodium dodecyl sulfate in 20 mM of Tris-buffered saline. This solution with and without the lens is respectively referred to as the TBS1X+lens and the TBS1X in the text.

Mechanical homogenization was achieved in all cases using a Precellys homogenizer (Bertin Technologies, France) with two rotation cycles at 6000 rpm separated by a 30-second pause. Only the last solution could be used on the D4$\sigma$ apparatus considering its transparency. The other solutions showed an inadequate degree of diffusivity readily observable with the eye (not shown). Noteworthy, the aqueous humor could be directly deposited in the quartz cuvette with remarkable transparency in the visible range as well as in the $650-900\,nm$ spectral range. Consequently, the aqueous humor NL properties could be directly characterized using the D4$\sigma$ technique. It is important to note that the goal of the homogenization process was not to achieve a molecular-level solution, but to produce a temporarily stable suspension with sufficiently low scattering to permit quantitative D4$\sigma$ measurements. The successful TBS1X-based preparation which consists of a facilitator of protein separation by decreasing the amount of protein aggregates resulted in a markedly clearer medium than the other methods, enabling the reliable acquisition of BSRV curves.

The second procedure took advantage of the intrinsic viscosity of the crystalline lens medium as well as the vitreous humor. More precisely, the vitreous humor was encapsulated between two standard microscope glass slides as is, separated by a 4 mm spacer. Likewise, the crystalline lens was placed between two parallel microscope slides separated by a 2 mm spacer. In order to remove its intrinsic converging properties due to its refractive index gradient^28^, the lens was gently de-structured using a surgeon scalpel before positioning between the slides. We precise here that the lens and vitreous humor transparency are greatly affected once they are withdrawn from the eye and slightly manipulated. The related increase of diffusivity is not adapted to the D4$\sigma$ technique. Thus, the phase object imaging method was employed on these samples to experimentally determine the NL refractive $n_2$ over the $650-900\,nm$ spectral range.

### D4$\sigma$ technique

**Fig. 5:**
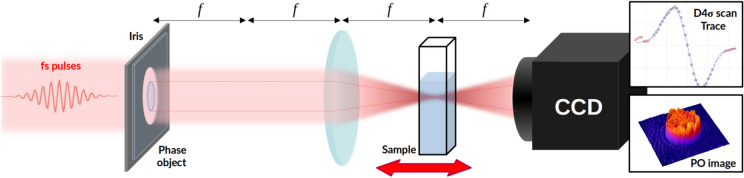
Diagram of Z-scan bench to determine the nonlinear index of a sample, by shifting it along the beam propagation axis.

The BSRV method (or D4$\sigma$) is a Z-scan-derived technique for measuring the NL refractive index $n_2$^16^. Unlike classical Z-scan, which monitors transmittance changes, BSRV quantifies the relative change in laser beam size as a sample is translated through the focal plane of a lens. This spatial measurement isolates beam narrowing or broadening–caused by self-focusing or defocusing–from confounding effects such as NL absorption. The resulting beam-width vs. sample position curve exhibits a characteristic dip whose amplitude and shape are directly related to $n_2$. By fitting the experimental curve to a beam propagation model in the NL medium and the optical system, $n_2$ can be extracted accurately. The method is especially valuable for characterizing transparent or weakly absorbing materials–including optical glasses, polymers, liquids, and biological tissues–when a clear separation between NL refraction and absorption is required. We note here that all the investigate media did not show any detectable absorption within our experimental conditions. In our configuration, a single lens images the diaphragm plane onto the CMOS camera, in a pseudo-4f set-up so that the beam profile is recorded during the sample displacement (see Fig. 5). The beam width is determined from the images using the ISO-standard second-moment (D4$\sigma$) definition^49^, which provides a robust estimate even for slightly distorted or non-Gaussian profiles. The NL refractive index is then inferred from the BSRV curve once the linear beam parameters, sample thickness, and focal intensity are known. This approach offers high sensitivity and improved signal-to-noise ratio compared with classical closed-aperture Z-scan, while avoiding normalization artifacts.

The crystalline lens sample exhibited significant structural inhomogeneity. Translating the sample along the Z-axis in the BSRV method caused the focal spot to sample different microscopic regions with varying optical properties, resulting in excessively noisy and non-reproducible BSRV profiles. For this reason, the phase object imaging technique–which relies on a single static measurement without sample translation–proved to be a more reliable method for slightly diffusive media as detailed below.

### Phase object imaging technique

**Fig. 6:**
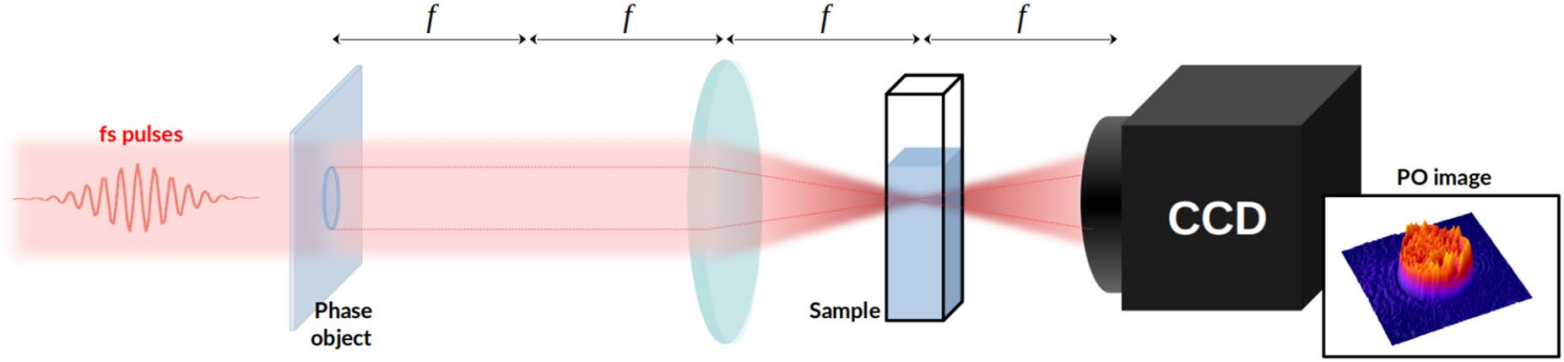
Experimental scheme for $n_2$ experimental determination using the phase object imaging technique in a pseudo-4f arrangement. By measuring the contrast in the phase object image (see inset) under NL irradiation and comparing it to a reference image obtained from a known medium (here water), the $n_2$ can be precisely evaluated.

The phase object imaging technique consists in quantifying the NL response occurring in the focal zone within the tested medium by analyzing the image of a well-known phase-only object as precisely described in^17^. When the phase object is positioned at a distance of 2*f* from a lens, its geometrical image is formed symmetrically at a distance of 2*f* on the opposite side of the lens, where a CMOS camera is placed for acquisition (see Fig. 6). The sample, optically thin as compared to the Rayleigh length, is located near the focal plane of the lens. In the linear regime, the image of the phase object is barely discernible and can only be inferred from the edges of the phase object with no contrast between the inner and outer part of the imaged phase object. However, when the illumination intensity is sufficiently high, a NL phase shift is induced, acting as a spectral phase filter. This NL interaction modifies the image, enhancing the contrast $c_m$ between the inner and the outer part of the imaged phase object as shown in the inset of Fig. 6. In that case, the total contrast $c_{m}$ of the medium is directly proportional to the NL induced phase shift accumulated by the laser pulse when propagating through the medium and the cuvette walls following:

$$c_{m} \propto \Delta \phi = \frac{2\pi I_{0}}{\lambda _0} \times \left( n_{2m} \cdot d + n_{2q} \cdot e \right)$$

where *d*= 10 mm is the medium thickness, $e=d/4$= 2.5 mm the cuvette total wall thickness and $I_{0}$ the laser intensity. By using the same cuvette of known $n_{2q}$ and identical laser intensities, the NL refractive index $n_{2m}$ can be inferred from the reference medium. We used water as a reference medium with $n_{2w}= 2 \times 10^{-20}\,m^2/W$ and yielding a contrast $c_w$. By calculating the contrast ratio $\frac{c_w}{c_m}$ one can obtain this practical formulae:

$$n_{2m} = n_{2w}\frac{c_m}{c_w}+\frac{n_{2q}}{4} \left( \frac{c_m}{c_w} -1 \right)$$

Advantageously, this expression doesn’t require a precise knowledge of $I_0$ which reduces the sources of uncertainties, provided that it remains sufficiently constant throughout the experiments. In practice, alternate measurements between water and other media were conducted to verify the experimental measurement stability and robustness. At least eight images were taken for each investigated wavelength and for each medium. The absolute uncertainty value can be estimated as $\Delta n_{2w} + \Delta n_{2q}$ that are provided from the D4$\sigma$ measurements. Thus the phase object imaging method presents a twofold higher degree of uncertainty than the D4$\sigma$ method.

## Conclusion

In this work, we have provided a consistent and quantitative characterization of the NL refractive index $n_2$ of three transparent media of the porcine eye–aqueous humor, crystalline lens, and vitreous humor–over the 650–900 nm spectral range. Using two complementary nonlinear optical techniques, namely the D4$\sigma$ method and the phase object imaging method, we demonstrated that all media exhibit comparable Kerr coefficients, with typical values on the order of $2 \times 10^{-20}\,m^2/W$ and only weak wavelength dependence within the investigated range. Furthermore, NL absorption was found to be negligible across the entire spectral range. The high level of agreement between the two techniques confirms the reliability of the measurements. These findings establish a robust reference set of $n_2$ values that can be directly implemented in numerical models of ultrafast laser propagation in ocular media.

Based on these measurements, we also evaluated the expected nonlinear propagation effects under conditions relevant to femtosecond cataract surgery. Our analysis shows that, although the Kerr effect does induce measurable phase and beam distortions, the magnitude of these effects remains moderate within clinically realistic focusing geometries.

This study provides essential experimental data for predictive modeling of light–tissue interaction in ophthalmic surgery and contributes to improving the control, safety, and effectiveness of femtosecond laser procedures. Future work may extend this approach to human tissues, to other spectral domains used in ophthalmology, and to dynamic measurements enabling the study of age-dependent or pathology-related variations of the nonlinear response.

Overall, our measurements provide a consistent set of nonlinear refractive indices for the main constituents of the anterior eye and indicate that, under standard femtosecond cataract-surgery conditions, both temporal and spatial nonlinear distortions remain limited. However, as emerging surgical platforms increasingly rely on spatial beam shaping, higher repetition rates, and multi-wavelength operation, a more comprehensive description incorporating Kerr, dispersive, and thermal effects–ideally through full NL Schrödinger equation modeling–will be essential to fully optimize precision and safety in ultrafast ophthalmic procedures.

## Data Availability

Datasets, graphs and original images are available from the corresponding author on request.
